# Horse and Cattle-Related Trauma: A Retrospective Review of Injuries and Management in a Regional Queensland Hospital

**DOI:** 10.7759/cureus.35746

**Published:** 2023-03-03

**Authors:** Gabriella Savage, Amanda Liesegang, Jakob Campbell, Matthew Lyon, Damian Fry

**Affiliations:** 1 General Surgery, Darling Downs Hospital and Health Service, Toowoomba, AUS

**Keywords:** rural surgery, injuries, trauma, horse, cattle

## Abstract

Background: Horse and cattle-related trauma is a common presentation to regional hospitals in Australia. We review local incidence and patterns of injuries relating to horse and cattle trauma over a three-year period at the Toowoomba Base Hospital within the Darling Downs region in Queensland, an area rich in cattle farming and equestrian recreation.

Methods: We conducted a single-centre retrospective cohort study. The inclusion criteria were all patients presenting with injuries following cattle or horse-related incidents between January 2018 and April 2021. Primary outcomes were the mechanism of trauma, confirmed injuries, and the need for admission, operative intervention, or inter-hospital transfer.

Results: A total of 1002 individuals (55% female; mean age 34 years; median Injury Severity Score (ISS) 2) were identified during the study period. Presentations relating to horses (81%) were more frequent than cattle (19%). The most common mechanism of injury was "falling" for horse incidents (68%) and "trampling" for cattle incidents (40%). Horse incidents often resulted in soft tissue injury (55%), upper limb fracture (19%), or lower limb fracture (9%). Cattle incidents often resulted in soft tissue injury (57%), upper limb fracture (15%), and rib fracture (15%). Overall, 14% required admission, 13% required operative intervention, and 1% required inter-hospital transfer.

Conclusions: This local series demonstrates a high volume of cattle and horse-related trauma in our region. Whilst most patients are managed locally without operative intervention, the high frequency of injuries observed necessitates further development of preventative measures and safety advocacy.

## Introduction

Cattle and horse-related trauma are frequent presentations to rural and regional hospitals in Australia [1-3], though there is a paucity of domestic literature describing the incidence and patterns of presentation or management. Injuries relating to these animals can be severe or even fatal, and some studies have even paralleled their severity to those seen in high-speed motor vehicle accidents [4,5]. However, most larger studies done internationally support that the majority of patients present with minor injuries and can be treated in local hospitals without the need for operative intervention or transfer to larger subspecialty trauma centers [2,6,7]. Data from tertiary trauma centers often fail to capture the true frequency and scope of injuries associated with cattle and horse-related incidents as the majority of presentations are managed regionally. Existing literature suggests that the mechanism of injury can directly relate to the pattern and severity of injuries [1,8], and there is some preliminary evidence that injuries can be prevented or reduced with the enforcement of general safety measures [2,4,9]. However, further appreciation of prevalence and description of patterns of presentation is required, to develop specific and effective public safety recommendations targeted at preventing these injuries. The Darling Downs Hospital and Southwest Health Services in Queensland support a vast region with remarkably high cattle density, as their geographic territories overlap with the Southern Fitzroy Basin (the largest cattle region in Australia with over 2.5 million head of cattle), the Queensland Murray Darling Basin (seventh largest cattle region in Australia), and the South West Basin (15th largest cattle region in Australia) [10]. Queensland overall has more than double the number of cattle than any other state in Australia [10]. Interspersed between the cattle farms are thoroughbred breeding properties, equestrian recreation centers, and private farming properties. Australia famously has the second-largest thoroughbred breeding industry in the world, and it is estimated that more than 31,000 Australian children per annum participate in equestrian sports and recreational activities [11,12]. As such, this study is uniquely positioned to describe the local incidence and patterns of presentations for cattle and horse-related trauma within a regional Australian health service densely surrounded by cattle farming and equestrian agriculture and recreation.

## Materials and methods

We conducted a single-centre retrospective cohort study including all patients who presented to Toowoomba Base Hospital Emergency Department with cattle or horse-related injuries from January 2018 to April 2021. Toowoomba Base Hospital is the main trauma referral centre for the Darling Downs Hospital and Health Service (DDHHS), and the Southwest Hospital and Health Service (SWHHS), located in regional Queensland, Australia. These health districts encompass over 480,000m^2^ of land and service a population of over 290,000 people combined [13]. Though Toowoomba Base Hospital is a level four trauma hospital, with the closest level one tertiary trauma centre being the Princess Alexandra Hospital in Brisbane (Queensland), it is the main trauma referral centre in this region due to the availability of surgical services, radiography including computerised tomography (CT) and magnetic resonance imaging (MRI), and an intensive care unit. Patients were identified by retrospectively searching the Emergency Department Information System (EDIS) database using the following keywords: “cattle”, “cow”, “horse”, “beast”, “pony”, “foal”, “colt”, “stallion”, “gelding”, “mare”, “filly”, “bull”, “heifer”, “steer”, and “yearling”. Each presentation was cross-referenced with clinical documentation to ensure suitability for inclusion. Patients with injuries that resulted from other livestock (i.e., pigs, dogs, sheep), or were only indirectly related to horses or cattle were excluded. Patient demographics, mechanism of injury, injuries, and management data were collected from clinical documentation accessed via The Viewer (Queensland Government Heath Provider Portal) and IMPAX (Agfa picture archiving and communication software). Data were grouped by age (paediatric vs. adult), gender (male vs. female), mechanism of injury (charged, kicked, fall, trampled, crushed, bitten), animal relating to the trauma (horse vs. cow), and management outcomes (discharged, admitted, underwent surgery, transferred to a tertiary centre). The Injury Severity Score (ISS) was calculated for all patients. Descriptive data analysis was performed using Microsoft Excel. The study was approved by the Darling Downs Hospital and Health Service Research Ethics Committee (HREC/2021/QTDD/75990) and therefore performed in accordance with the ethical standards laid down in the Declaration of Helsinki.

## Results

**Figure 1:**
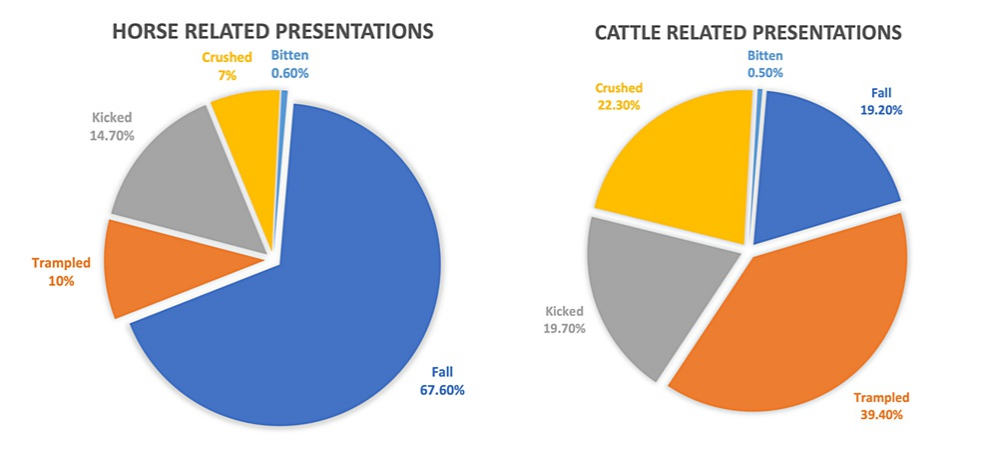
Mechanisms of injury for horse and cattle-related presentations grouped by animal

**Figure 2:**
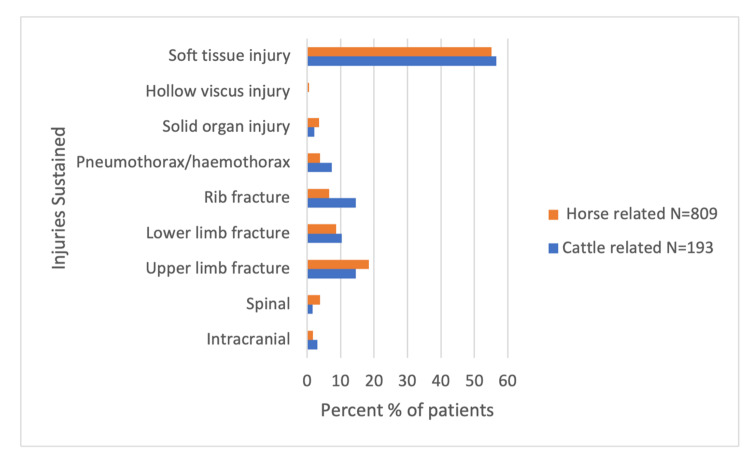
Patterns of injuries sustained following horse and cattle-related incidents grouped by animal type

Between January 2018 and April 2021, 1002 patients presented to the Toowoomba Base Hospital Emergency Department with horse or cattle-related trauma. There was a slight female preponderance (54.5%) and the mean age was 34 years (range 0 to 91). The average ISS for all patients was 2. A total of 80.7% of presentations were horse related, with falling (67.6%) and being kicked (14.7%) being the most common mechanisms (Figure 1). Horse-related incidents most frequently involved females (62.5%), and commonly resulted in soft tissue injury (55.1%), upper limb fracture (18.5%), and lower limb fracture (8.7%). Cattle injuries (19.3%) were more likely a result of being trampled (39.4%) or crushed (22.3%) (Figure 2). Cattle incidents more frequently involved males (79.3%), and commonly resulted in soft tissue injury (56.5%), rib fractures (14.5%), and upper limb fractures (14.5%). Cattle-related incidents resulted in more pneumothorax/haemothorax and intracranial injury, however, resulted in less frequent spinal injury (Figure 2). Further descriptions of injuries grouped by animal type are displayed in Table 1. Overall, the average ISS for cattle-related incidents was slightly higher than for horse-related incidents (2.3 compared to 1.9). There were 203 paediatric patients (aged 16 or under), 20.3% of the overall patient cohort. A majority of paediatric patients were female (72.9%) and the median age was 12. Over 90% of paediatric injuries occurred because of a horse, with fall being the most reported mechanism (77.3%). Soft tissue injury (47.3%) and upper limb fracture (31.0%) were the most prevalent injuries, and no paediatric patients sustained rib fractures or pneumothorax/haemothorax. Further description of injuries grouped by paediatric/adult subgroups is displayed in Table 2. The average ISS for paediatric presentations was slightly higher when compared to adult presentations (2.6 compared to 2). Of all patients, 74.4% were discharged directly from the emergency department (ED), and 24.5% were admitted, with similar proportions for both paediatric and adult groups. When comparing horse and cattle groups, cattle-related presentations were more likely to be admitted and more likely to require operative intervention (Table 1). Twelve individuals (1.2%) required transfer to a major tertiary centre, and one individual died as a direct result of their injuries. This female patient aged 70s presented to the hospital three days after being kicked by a horse and died because of intra-abdominal sepsis, secondary to a bowel injury.

**Table 1:** All patients presenting with horse and cattle-related injuries between January 2018 and April 2021 grouped by animal type

		Cattle-related presentations N = 193	Horses-related presentations N = 809
Gender	Female	40/193 (20.7%)	506/809 (62.5%)
	Male	153/193 (79.3%)	303/809 (37.5%)
Age	<16 years (N=203)	-	-
	Female	3/18 (16.7%)	145/185 (78.4%)
	Male	15/18 (83.3%)	40/185 (21.6%)
	>16 years (N=799)	-	-
	Female	37/175 (21.1%)	361/624 (57.9%)
	Male	138/175 (78.9%)	263/624 (42.1%)
Mechanism of trauma	Fall	37/193 (19.2%)	547/809 (67.6%)
	Trampled	76/193 (39.4%)	81/809 (10.0%)
	Kicked	36/193 (18.7%)	119/809 (14.7%)
	Crushed	43/193 (22.3%)	57/809 (7.0%)
	Bitten	1/193 (0.5%)	5/809 (0.6%)
Confirmed injuries	Intracranial	6/193 (3.1%)	14/809 (1.7%)
	Spinal	3/193 (1.6%)	31/809 (3.8%)
	Upper limb fracture	28/193 (14.5%)	150/809 (18.5%)
	Lower limb fracture	20/193 (10.4%)	70/809 (8.7%)
	Rib fracture	28/193 (14.5%)	53/809 (6.6%)
	Pneumothorax/haemothorax	14/193 (7.3%)	31/809 (3.8%)
	Solid organ injury	4/193 (2.1%)	29/809 (3.6%)
	Hollow viscus injury	0	5/809 (0.6%)
	Soft tissue injury	109/193 (56.5%)	446/809 (55.1%)
Injury Severity Score	Mean (range)	2.3 (0-16)	1.9 (0-18)
Management	Emergency department discharge	122/193 (63.2%)	623/809 (77%)
	Admission	69/193 (35.8%)	176/809 (21.8%)
	Operative intervention	37/193 (19.2%)	104/809 (12.9%)
	Inter-hospital transfer	2/193 (1%)	10/809 (1.2%)

**Table 2:** All patients presenting with horse and cattle-related injuries between January 2018 and April 2021 grouped by age

Demographics		All patients N = 1002	<16 years N = 203	>16 years N = 799
Gender	Female	546/1002 (54.5%)	148/203 (72.9%)	398/799 (49.8%)
	Male	456/1002 (45.5%)	55/203 (29.1%)	401/799 (50.2%)
Age	<16 years	203/1002 (20.3%)	-	-
	>16 years	799/1002 (79.7%)	-	-
Trauma-related to	Horse	809/1002 (80.7%)	185/203 (91.1%)	624/799 (78.1%)
	Cattle	193/1002 (19.3%)	18/203 (8.9%)	175/799 (21.9%)
Mechanism	Fall	584/1002 (58.3%)	157/203 (77.3%)	427/799 (53.4%)
	Trampled	157/1002 (15.7%)	18/203 (8.9%)	139/799 (17.4%)
	Kicked	155/1002 (15.5%)	19/203 (9.4%)	136/799 (17.0%)
	Crushed	100/1002 (10.0%)	7/203 (3.4%)	93/799 (11.6%)
	Bitten	6/1002 (0.6%)	2/203 (1.0%)	4/799 (0.5%)
Confirmed injuries	Intracranial	19/1002 (1.9%)	4/203 (2.0%)	15/799 (1.9%)
	Spinal	34/1002 (3.4%)	2/203 (1.0%)	32/799 (4.0%)
	Upper limb fracture	178/1002 (17.8%)	63/203 (31.0%)	115/799 (14.4%)
	Lower limb fracture	90/1002 (9.0%)	8/203 (3.9%)	82/799 (10.3%)
	Rib fracture	79/1002 (7.9%)	0	79/799 (9.9%)
	Pneumothorax/haemothorax	45/1002 (4.5%)	0	45/799 (5.6%)
	Solid organ injury	32 (3.2%)	1/203 (0.5%)	31/799 (3.9%)
	Hollow viscus injury	5 (0.5%)	0	5/799 (0.6%)
	Soft tissue injury	554 (55.3%)	96/203 (47.3%)	458/799 (57.3%)
Injury Severity Score	Mean (range)	2 (Range 0-18)	2.6 (Range 0-16)	2 (Range 0-18)
Management	Emergency department discharge	745/1002 (74.4%)	159/203 (78.3%)	586/799 (73.3%)
	Admission	245/1002 (24.5%)	42/203 (20.7%)	203/799 (25.4%)
	Operative Intervention	141/1002 (14.1%)	29/203 (14.3%)	112/799 (14.0%)
	Inter-hospital transfer	12/1002 (1.2%)	2/203 (1%)	10/799 (1.3%)

## Discussion

This study highlights the prevalence of horse and cattle-associated traumatic injuries within a large regional area with high cattle and equestrian density. We demonstrate a high frequency of trauma presentations relating to horses and cattle, observing just over six individual presentations per week at our facility. Whilst similar volumes of presentation have been observed in select international studies [14], the majority of existing literature includes far smaller patient cohorts despite similar periods of observation [5]. This can be attributed to some environmental factors such as the density of horsing and cattle activity surrounding specific hospital services, as well as the presence and proximity of other hospitals or trauma centres to which patients may be preferentially transported. The high volume of data captured in this study may also be explained by the inclusion criteria, which unlike most of the existing literature, includes all presentations to the ED irrespective of eventual admission or discharge. Our data support existing literature that overall horse-related injuries are much more common than cattle-related injuries (80.7% and 19.3% respectively) [6,8]. We also demonstrate a strong female prevalence in horse-related injuries overall (62.5%), consistent with existing studies [1,15]. Acton et al. [15] observed in a large 27-year multicentre American study that more than 63% of horse-related trauma presentations were female. However, it is important to note that the female prevalence in horsing injuries is likely partially skewed by paediatric data. In our dataset, whilst females accounted for 78.4% of horse-related paediatric incidents, females accounted for only 57.9% of horse-related injuries in adults. Lang et al. [1] also describe the marked gender discrepancy in the paediatric population in a study of paediatric horse-related injuries in Queensland, Australia, where they reported that 72.4% of the children injured in horse riding were female, and only 49.2% of their injured adults were female. This higher proportion of horse-related injuries in female children may be explained by overall higher rates of participation in organised equestrian and horse-riding activities, with just 0.3% of Australian males participating in equestrian sports compared to 2.0% of females, according to Australian Census Data. The discrepancy in equestrian participation between genders is less pronounced in adults [11]. Conversely, there is a strong male predominance in cattle-related injuries overall (79.3%), irrespective of age. Murphy et al. [8] in 2010 reviewed cattle-related trauma presentations to a regional hospital in Ireland and found more than 74% of patients were male, echoed by Watts et al. [7] in 2011. Just as female children may dominate participation in equestrian recreation, anecdotally male children are more likely to be involved in recreational cattle activities such as poddy calf riding. With regards to adults, there has traditionally been a much higher proportion of male adult individuals working in cattle farming and agricultural communities, though the proportion of female agricultural workers is slowly increasing according to recent reports by the Australian Department of Agriculture [16]. Our participants were most likely to be injured by a horse when falling from riding, or being kicked, consistent with existing literature [15]. As such, the injury patterns observed were frequent upper limb (18.5%) and lower limb (8.7%) fractures. It is thought that this may occur as upper and lower limbs are often reflexively positioned to break falls, especially from a horse’s height. Spinal injury, though infrequent, was seen more frequently in horsing injuries than in the cattle cohort, and it is thought that this may relate to the potential for axial loading or hyperflexion when falling from height at speed. With regards to cattle, trampling or crush mechanisms were most common, also observed by Rhind et al. [5] and Murphy et al. [8]. Whilst limb fractures are still seen following cattle-related incidents, rib fractures, pneumothorax/haemothorax, and intra-cranial injury were seen more frequently than in the horse group. It is felt that this corresponds with the blunt force exerted towards the thorax or skull, as would be seen in trampling, or crushing mechanisms [8].There was no obvious discrepancy with regard to hollow viscus injury or solid organ injury between our animal groups. Though limb fractures were common in our paediatric group, no paediatric patients sustained rib fractures. It is well understood that rib fractures are exceedingly rare in paediatric trauma due to increased chest wall compliance [17]. Overall, the severity of injuries in our cohort was low, as our average ISS was 2. This was only marginally higher in those presenting with cattle-related injuries (ISS 2.6). This contrasts with other reports with average ISS as high as 13-17 [5] in cattle-related trauma, and 10 [4] in horse-related trauma. A possible explanation for an overall low ISS in our cohort of patients is that individuals with higher ISS scores may have been primarily retrieved from the site of injury and transferred directly to a tertiary trauma centre in order to receive input from subspecialist input such as neurosurgery or interventional radiology services. Those with complex injuries likely to require neurosurgery, interventional radiology, or multi-subspeciality input, often bypass our hospital as we do not provide these services. Additionally, some studies such as Lang et al. [1] only calculated ISS scores for those admitted to the hospital, whilst our report includes all presentations to ED. Most presentations (74.4%) were discharged from the ED, and 24.5% required admission. Patients with cattle-related injuries were more likely to be admitted, and more likely to require operative intervention, likely reflecting the type and severity of injuries. Of those admitted, 54% of our cattle group required surgery, similar to reports by Murphy et al. [8] and Rhind et al. [5] who published rates of operative intervention of 50-60%. Whilst patients with horse-related injuries were less likely to require an operation if they presented to ED, the rate of requiring surgery if they were admitted was 60%, similar to Lang et al. [1] who observed 54% of their patients require surgery. Though existing studies did not comment on the requirement for interhospital transfer in their cohorts, it is pertinent to note given our report was conducted in a regional centre with limited trauma capabilities. We found no difference in our rate of requiring interhospital transfer between our cattle and horse groups, or between our adult and paediatric groups. Overall, only 1.2% of patients are required to transfer from our centre. This reflects that appropriate triaging and bypassing algorithms are being used by our primary retrieval services (critical care paramedics and doctors that attend trauma incidents) and suggests our health service’s capability in managing a high volume of mild-moderate trauma despite our peripheral geographic location. Our study emphasises the volume of horse and cattle-related trauma presented to a regional Australian hospital. Whilst horse-related injuries are more common, cattle-related injuries tend to be more severe. As patterns of mechanisms and injury are specific to the animals, strategies for protection and prevention should be approached as such. Associations such as Horse Safety Australia Inc. [18] provide site accreditation assessments aimed at regulating safety features at facilities with horses and run education clinics in order to certify those in the equestrian industry for safe horse handling. This is particularly pertinent as over 90% of horse-related injuries occur in recreational or sporting settings [19]. Safety products such as helmets and breakaway stirrups are encouraged, though have not been regulated. Safe Work Australia and Australian Agricultural Health and Safety have published guides on safe cattle handling practices [12,20], which include relevant information on minimising risk around cattle handling by optimising the environment and individual education. There are no specific personal protective equipment recommendations for cattle handling, however, and no regulated accreditation processes for cattle handling facilities. Based on our findings of frequent rib fractures and soft tissue injuries, we query if protective padded clothing may be beneficial for cattle handlers. Ultimately, a further in-depth understanding of how these injuries occur is required in order to develop specific and relevant recommendations or regulations for prevention. Our clinical data is limited to the information that is collected in the ED, which does not include incidental analysis.

## Conclusions

This review has demonstrated a high volume of cattle and horse-related trauma in a small regional health service surrounded by cattle farming and equestrian agriculture and recreation. Whilst most injuries were minor and patients are managed locally without operative intervention, the high frequency of injuries observed necessitates further development of preventative measures and safety advocacy. We would advocate for a centralised reporting system. This would allow formal reporting of incidents and collating details surrounding their occurrences so that more detailed risk factors for injuries can be understood so that meaningful recommendations can be made, and these injuries may be better understood and therefore prevented.
